# Explainable text-tabular models for predicting mortality risk in companion animals

**DOI:** 10.1038/s41598-024-64551-1

**Published:** 2024-06-20

**Authors:** James Burton, Sean Farrell, Peter-John Mäntylä Noble, Noura Al Moubayed

**Affiliations:** 1 Department of Computer Science, Durham University, Durham, UK; 2 Institute of Infection, Veterinary and Ecological Sciences, University of Liverpool, Liverpool, UK; 3 Evergreen Life Ltd, Manchester, UK

**Keywords:** Computer science, Machine learning

## Abstract

As interest in using machine learning models to support clinical decision-making increases, explainability is an unequivocal priority for clinicians, researchers and regulators to comprehend and trust their results. With many clinical datasets containing a range of modalities, from the free-text of clinician notes to structured tabular data entries, there is a need for frameworks capable of providing comprehensive explanation values across diverse modalities. Here, we present a multimodal masking framework to extend the reach of SHapley Additive exPlanations (SHAP) to text and tabular datasets to identify risk factors for companion animal mortality in first-opinion veterinary electronic health records (EHRs) from across the United Kingdom. The framework is designed to treat each modality consistently, ensuring uniform and consistent treatment of features and thereby fostering predictability in unimodal and multimodal contexts. We present five multimodality approaches, with the best-performing method utilising PetBERT, a language model pre-trained on a veterinary dataset. Utilising our framework, we shed light for the first time on the reasons each model makes its decision and identify the inclination of PetBERT towards a more pronounced engagement with free-text narratives compared to BERT-base’s predominant emphasis on tabular data. The investigation also explores the important features on a more granular level, identifying distinct words and phrases that substantially influenced an animal’s life status prediction. PetBERT showcased a heightened ability to grasp phrases associated with veterinary clinical nomenclature, signalling the productivity of additional pre-training of language models.

## Introduction

Life expectancy serves as a fundamental metric for understanding human and animal populations’ overall health and well-being^1^. Understanding life expectancies permits insights into the health status of a populace and aids in the identification of health disparities and inequalities between specific regions. Tools designed for monitoring mortality play a vital role in assisting researchers in pinpointing events occurring earlier in life that may reduce overall lifespan. Nevertheless, national mortality rates for companion animals are not subject to regular monitoring. The surveillance of electronic health records (EHR) collected from primary-care veterinary practices represents a valuable means to gain insights into companion animals’ current population health status. Initiatives such as the Small Animal Veterinary Surveillance Network (SAVSNET) have played a pivotal role in establishing accessible, real-time, first-opinion clinical EHRs on a national scale in the United Kingdom^2^. Despite their potential, it is challenging to harness the total utility of first-opinion veterinary EHRs on a large scale. The implementation of disease coding frameworks, while advantageous for researchers, often proves counter-intuitive in clinical practice and impractical for everyday use. Previous studies have underscored records annotated by clinicians as part of their routine responsibilities as being particularly susceptible to inaccuracies and omissions^3,4^. Adopting an unstructured, free-text format in contemporary veterinary EHRs while affording clinicians greater linguistic flexibility presents challenges in developing automated systems^5,6^. Moreover, veterinary practices typically do not have dedicated staff for disease coding, resulting in distinct naming conventions and practice-specific clinical narrative structures, thereby needing more harmonisation in recording clinical variables. In response to these challenges, a pressing need exists to establish fixed, tabular data points for clinical events that do not impose additional complexity on clinicians’ responsibilities whilst facilitating downstream data analysis.

Recent advancements in Natural Language Processing (NLP) have significantly improved a wide range of text-driven tasks. A pivotal breakthrough in this domain is the integration of the transformer architecture, featuring the self-attention mechanism^7^. This architectural paradigm was initially realised in the Bidirectional Encoder Representation for Transformers (BERT)^8^, marking the dawn of a new era characterised by state-of-the-art performance in various NLP benchmarks^9,10^. Integrating such language models into the analysis of clinical narratives is not a novel concept^11^. Prior research has demonstrated their efficacy in a diverse array of NLP tasks, including relation extraction^12^, named entity recognition^13,14^, and sequence classification for disease coding^15,16^. Similarly, for tabular data, there exists a diverse set of methodologies, such as gradient boosting methods, including XGBoost^17^, and deep learning approaches, such as TabNet^18^. These, too, have been explored for a diverse set of tasks such as prediction-based modelling for hypertension^19^ and survival analysis^20^. The utilisation of deep learning modelling within the clinical domain has been widely explored, with examples including intensive care patient management^21^, hospital mortality prediction^22^, predicting infections such as COVID-19^23^ and sepsis^24^, and decisions related to performing medical procedures such as when to mechanical ventilate^25^, among others.

However, a significant obstacle to trusting the results from deep learning models for use within a clinical setting is their lack of explainability, preventing much of the above research from leaving the proof-of-concept stage. Many associated challenges, including bias mitigation and the generalisability of these models, can be traced back to the fundamental issue of incomplete model interpretability^26^. These issues are further exacerbated when considering data sources from multiple modalities, such as text and tabular data, for use within machine learning frameworks. Nonetheless, these distinct data types frequently harbour a wealth of intricate signals where, in some instances, consolidating these data types can enhance overall predictive accuracy as they synergistically complement each other, resulting in a more robust and comprehensive prediction model. In the clinical domain, we find compelling instances of multimodal models that have harnessed this diversity. For instance, in the identification of cataract cases^27^, innovative approaches have combined free-text electronic medical records, structured tabular data, and scanned clinical images, resulting in a notable enhancement in model performance relative to their single modality counterparts. Where deep learning model studies have been designed to leverage available patient data, combining free-text notes from medical professionals with tabular data points, the already limited arsenal of conventional explainability frameworks, such as LIME^28^ or SHAP^29^, is insufficient, as they can only address one modality at a time. Consequently, a compelling need arises to develop comprehensive explainability systems for deep learning models in clinical contexts that capture all the available data and ensure that healthcare practitioners make clinical decisions with confidence and trust.

This paper builds upon our previous research by applying a novel multimodal masking framework that extends the applicability of SHapley Additive exPlanations (SHAP) to text-tabular datasets within the veterinary clinical domain^30^. We apply a range of combination methodologies within the framework to merge these differing modality types and, in doing so, outperform the previously proposed methodology. Through feature masking based on their respective modalities, our framework ensures consistent treatment of features, fostering predictability in unimodal and multimodal contexts. In this study, we apply our framework to a dataset of EHRs sourced from first-opinion veterinary practices across the UK. Our research explores a comparative analysis, highlighting the effectiveness of additional fine-tuning processes, exemplified by PetBERT - a large language model tailored to veterinary clinical records. Our findings demonstrate its superior performance over general corpora models like BERT-base in tasks specific to our domain. The investigation delves into the unique words, phrases and individual tabular values to directly compare which characteristics significantly affect the prediction of an animal’s living status. By incorporating multiple modalities, including breed, age, deprivation scores, and clinical narratives, we unveil the features contributing to increased mortality risk. The implications of this research extend beyond animal welfare, highlighting the potential of a multimodality explanation framework applicable across diverse tasks.

## Background

### Dataset

Electronic health records have been collected since March 2014 by SAVSNET, comprising a sentinel network of 253 volunteer veterinary practices found across the United Kingdom. A full description of SAVSNET has been presented elsewhere^2^. Generally, veterinary practices with practice management software compatible with the SAVSNET data exchange are recruited based on convenience. Within these participating practices, data is collected from each booked consultation (where an appointment has been made to see a veterinary practitioner or nurse). All owners attending these practices can opt out of data collection at the time of consultation. Data is collected on a consultation-by-consultation basis and includes information such as species, breed, sex, neuter status, age, owner’s postcode, insurance and microchipping status and, crucially to this study, a free-text clinical narrative outlining the events that occurred within that consultation. Appended to all the SAVSNET EHR datasets are high-level International Classification Disease 11 (ICD) codings. These syndromic labels can provide a broad overview of the themes within the clinical narrative, a free-text field. A full explanation of how these were derived is explained elsewhere^31^. Sensitive information, such as personal identifiers, was cleaned from the data. SAVSNET has ethical approval from the University of Liverpool Research Ethics Committee (RETH000964).

### BERT and PetBERT

BERT-base was previously pre-trained on a combination of Wikipedia and BookCorpus^8^. During pre-training, BERT-base performed two tasks simultaneously: Masked Language Modeling (MLM) and Next Sentence Prediction (NSP). In the MLM task, words within a sentence were randomly replaced with a [MASK] token, with a 15% probability across the entire dataset. The model’s objective was to predict the original or a similar word in place of the [MASK] token. For the NSP task, sentences were randomly split and combined either with the original sentence or with a random sentence, separated by a [SEP] token. The goal was to determine if the combined sentences made sense or not.

PetBERT was formed by taking the pre-trained BERT-base model and undertaking additional pre-training (also performing MLM and NSP) on a large dataset of over 500 million tokens from the SAVSNET first opinion veterinary corpus, exposing it to clinical language used in veterinary contexts. A more detailed explanation of the training process can be found elsewhere^31^. It is these pre-trained BERT and PetBERT models that will be fine-tuned on the classification task in the subsequent analysis.

### Multimodal SHAP

To uncover the most important features for the model to predict the animal’s given mortality risk, we employ our novel multimodal SHAP that was first introduced in^30^. Using this tool, we can produce SHAP explanations for the text-tabular SAVSNET dataset for the first time. SHAP is a game theory-based technique based on simulating the presence and absence of coalitions of features to assess the impact on the outcome variable^29^. The original, unimodal SHAP library is limited to explaining one modality at a time. For text, absent features are replaced with a [MASK] token, whereas tabular features—where “empty” or N/A values are not always modelled—are simulated as absent by sampling from a background dataset and integrating over the marginal distribution. When using unimodal SHAP, the only time it is feasible to generate explanations from a multimodal dataset is when the input is preformed into a single modality: text. However, this leads to the problematic grouping of features and importance assigned to ever-present, non-feature input. Multimodal SHAP^30^ brought the two approaches into a single framework so that text and tabular features are treated distinctly, consistent with how they would be in an unimodal scenario. This enables the direct comparison of words and phrases within the text features against tabular features for any method of combining the two modalities.

## Methods

### Data extraction

To curate the datasets for training the initial component of the predictive mortality models, we searched for narratives containing references to death or euthanasia. This search used a generalised Python regular expression to identify pertinent terms, including “euthanasia”, “put to sleep (PTS)”, and “died”. The detailed regex pattern is provided below.

*euth|dead|died|pts|put to sleep|pento|doa|crem|burial|bury|qol|quality|ashes|scatter|casket*

Subsequently, from this dataset, we performed random sampling to select 250 cases that were suspected to involve mentions of death or euthanasia. These selected cases underwent manual inspection to validate whether they conformed to the predefined case definition of “declaration of death occurring within the consultation”. Notable instances of false positives included conversations of potential future euthanasia events or instances where euthanasia was discussed in an advisory context by the attending practitioner. Instances where the euthanasia event did not occur within the same consultation were excluded or used as the controls in equal proportion to the number of cases. The EHR data used in this study offers valuable insights into death occurrences among the dogs and cats analysed. However, the depth of our analyses is contingent upon the information recorded by the veterinary practitioners. Consequently, our models are limited to capturing only those conditions or events explicitly documented in the EHRs. Any unrecorded or overlooked aspects cannot be accounted for in our analysis.

**Table 1:** Each of the 31 variables from the SAVSNET dataset that are used in this analysis.

Variables included in the analysis	
Clinical narrative (written by veterinary clinician)	
General features:	
Age at consult; Breed; Species; Gender; Insured status; Neutured status; Region (of owner’s postcode); Practice ID; Premise ID	
Indicators of a disease, a condition or disorder involving:	
Circulatory system; dental; development; digestive system; endocrine, nutritional or metabolic disorders; immune system; neurodevelopment; infectious or parasitic diseases; skin; musculoskeletal or connective tissue; visual system; perinatal conditions; pregnancy, childbirth or puerperium; ears; blood-forming organs; respiratory system; injuries; poisoning or external causes; genitourinary system; neoplasms; nervous system	

A semi-supervised teacher-student model approach was adopted in line with the methodology employed by Yalniz et al.^32^. This approach used a small subset of manually annotated records to train a small binary sequence classification model, which achieved an F1 Score of 98.3% on the test set. This model was subsequently applied to the entire dataset to identify animals meeting the criteria. To ascertain the effectiveness of this extraction method, a random sample of 200 records was independently reviewed by a practising clinician to validate the model’s performance and suitability for the continuation of the study. For the animals identified to have died by the aforementioned binary sequence classification task, we took the consultation preceding the declaration of death. To create a balanced dataset, animals stated to be alive by the binary sequence classification model were pulled at equal quantities to the number of animals that had died. Narratives for cases where the animal had only a single narrative in its history (the one detailing its death) were discarded. Within both the case and controls, where incomplete data exists, such as missing breed, age, sex, geographical information, or where an animal appeared in both case and control datasets, these records were also deleted. All high-level ICD codings that the animal has previously amassed were summed together. We used the frequencies of each ICD coding to represent each animal’s approximate clinical history and maximise the availability of tokens available for the penultimate clinical narrative for PetBERT. The dataset contains many tabular features, such as age, breed, and sex, which were likely to play a role in supporting the model’s prediction capabilities. Features used in this study are found in Table 1.

Datasets were split into training and testing based on an 80:20 split. The 20% of records used for the testing set was pulled from $\approx$2.1 million annotated records that were not used in the initial pre-training of PetBERT^31^. This ensured the model’s weightings were generalisable to all first-opinion clinical narratives, so no element of these testing sets had been pre-exposed to the model in any form.

**Figure 1:**
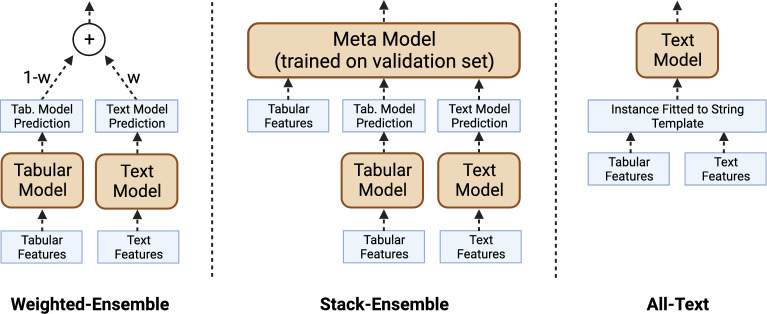
Combination methods used in this study as proposed in the original methodology^30^. Left: a *Weighted-Ensemble*. Middle: a *Stack-Ensemble*. Right: *All-Text*.

### Model training

We determine whether an animal known to have died within 28 days of the last given consultation can be identified using all five combination methods used in the original study^30^, first with BERT as the language model and then repeat with PetBERT as the language model, for a total of 10 experiments. SAVSNET data contains a mixture of free-text and tabular features, so in this study, we utilise five different methods of training a model with both modalities. Figure 1 outlines the combination methods. Following the original study, we use the *All-Text* approach, three *Weighted-Ensembles* models and a *Stack-Ensemble*. In the *All-Text* approach, all features are fit to a string template and fed to a large language model; we use the same format as in Multimodal SHAP^30^, namely: *Column name 0: Column value 0 | Column name 1: Column value 1 | ...*. For the *Weighted-Ensembles*, text and tabular models are trained separately, and their predictions are combined in weighted sum, with *w* as the weight of the text model prediction and *1-w* as the weight of the tabular model prediction). We experiment with three values of *w*: 0.25, 0.50 and 0.75. The *Stack-Ensemble* method also requires separately trained text and tabular models but also has a third model, a meta-model, trained on the tabular features and the text and tabular predictions using the validation set. To avoid extensive one-hot encoding, or ordinally encoding variables with no linear relationship, we treat features with more than 30 unique values as text features; specifically, this was utilised for the breed and region features. These are conjoined with the free-text clinical narrative using the same text template outlined in the *All-Text* method. The aim is to understand which features, whether words or phrases within the free-text clinical narrative, or numerical or categorical entries within the tabular data, are important in making a prediction. All tabular and meta-models are light gradient boosting classifiers^33^.

### Model evaluation

For each text model-combination method pair, we evaluate the performance against a test dataset selected from the 2m records set aside from the initial pre-training of PetBERT. Therefore, this test set contained records that had not been seen by PetBERT in either the initial masked learning step or in the downstream classifications step. Values were compared using the animals’ true mortality outcome as a baseline result. Following the original PetBERT paper^34^, we report performance using the F1 score, see Table 2. For added information, we also report accuracy in Table 3.

**Table 2:** Test set F1 scores for all models.

	Stack	WE *w*=0.25	WE *w*=0.50	WE *w*=0.75	All Text	All Text (Txt fts only)	Tab Model
Petbert	0.828	0.823	**0.844**	0.828	0.831	0.811	0.802
BERT	0.821	0.823	**0.828**	0.800	0.823	0.783	0.802

We also report the scores when only the text columns are used to train the language models, labelling this All Text (Txt fts only).

* WE* Weighted-Ensemble.

Significant values are in bold.

**Table 3:** Test set accuracy scores for all models.

	Stack	WE *w*=0.25	WE *w*=0.50	WE *w*=0.75	All Text	All Text (Txt fts only)	Tab Model
Petbert	0.820	0.810	**0.832**	0.814	0.823	0.794	0.789
BERT	0.807	0.810	**0.817**	0.785	0.816	0.766	0.789

We also report the scores when only the text columns are used to train the language models, labelling this All Text (Txt fts only).

* WE* Weighted-Ensemble.

Significant values are in bold.

### Generating SHAP values

Our goal is to explore the reasons for the similarities and differences in the performances and investigate why PetBERT outperformed BERT for each of the five combination methods. To do so, we generate SHAP values for each combination of the two independent variables: combination method (CM) and text model (TM). To isolate the differences in explanations to the independent variables, we choose the same 1000 randomly selected test-set examples to be explained for each TM-CM combination.

## Results

To account for the variations observed in label counts and token quantities across all instances, we utilise a process similar to those developed within the original SHAP package’s summary plot function. Whereas tabular features produce a single SHAP value, text features produce a SHAP value for each word piece. Therefore, we sum the SHAP values. Specifically, there are $T$ tokens for each instance, each belonging to one of $F$ features. Each token has associated SHAP values for $L$ labels, which for this binary classification task is 2. First, the SHAP values for each token are summed, $t\in {T}$, belonging to a feature, $f\in {F}$ before converting to the absolute value and sum across each of the two labels, $l\in {[\text {alive, dead}]}$. Therefore, a single SHAP value for each feature in each instance indicates how important the feature was to the model. We refer to this as feature importance or $\phi$.

1 $$\begin{aligned} \phi _{f} = |\sum _{t\in {f}}\text {SHAPvalue}_{t,l=\text {alive}} |+ |\sum _{t\in {f}}\text {SHAPvalue}_{t,l=\text {dead}} |\end{aligned}$$

### Which features are the most important?

**Figure 2:**
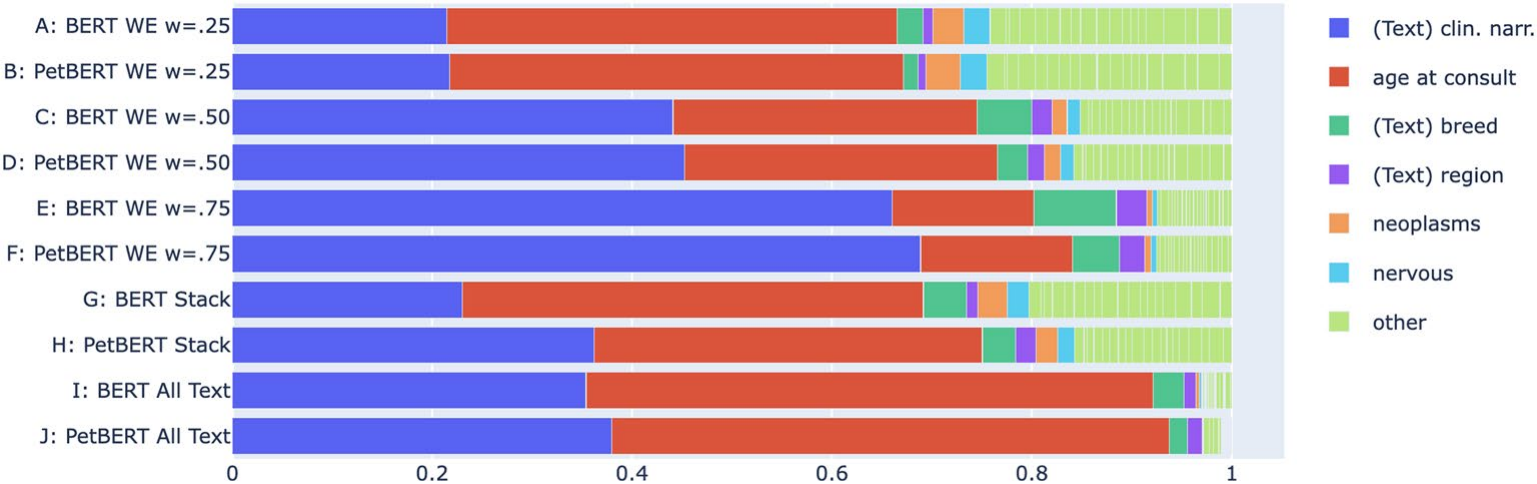
Mean absolute feature importance, by feature, as a proportion of the sum of all mean absolute feature importance. Each row indicates the proportion for a particular model, as indicated on the y-axis. We look across all experiments to find the six most influential features, colouring the remaining features as lime-green in an *other* category. The size of each coloured bar indicates the feature’s relative overall importance to the particular model, with cumulative proportion on the x-axis. The order of the colours is the same for each row, in order of highest to lowest proportion across all experiments.

Typically, to see how important a feature is across the entire dataset, one would use a SHAP summary plot, which shows mean absolute SHAP values for each feature, averaged across the entire dataset. In our analysis, we use mean absolute $\phi$. To compare across experiments, for each TM-CM pair, we plot mean absolute $\phi$ as a proportion of the sum of all mean absolute $\phi$ for that pair. This is shown in Fig. 2.

For each of the three *Weighted-Ensembles*, we see a linear increase in the reliance on textual features as *w*, the text model weighting, increases, a pattern we see for both BERT and PetBERT. When *w* = 0.25, we see *age at consult* as the most important feature overall, whereas for *w* = 0.50 and 0.75, *clinical narrative* is the most important. The *Stack-Ensembles* follow the pattern of PetBERT relying on *clinical narrative* more than BERT, however with *age at consult* as the most influential feature. The *All-Text* models represent the only experiments where tabular features are fed into a text model. These results demonstrate that despite this, language models can indeed extract use out of tabular features with both PetBERT *All-Text* and BERT *All-Text* focusing on *age at consult* the most.

### How similarly are features ranked?

In this section, we look at how statistically similar the rankings of features are for a given instance. To do so, we use Kendall’s rank correlation coefficient^35^, or Kendall’s $\tau$. This is a non-parametric test that does not consider the size of a particular value but simply the relative ranking, making it suitable for comparison across different methods and models. An identical ranking of features will score 1, and an opposite ranking will score $-1$.

2 $$\begin{aligned} \tau = \frac{\text {Number of concordant pairs} - \text {Number of discordant pairs}}{\text {Total number of pairs}} \end{aligned}$$

In Fig. 2, results were averaged across all instances and then reported. Here, we calculate Kendall’s $\tau$ for each instance and then average, reporting the mean and standard deviation of the statistic to facilitate a more nuanced examination. In Tables 4, 5 and 6, for a particular comparison, we will calculate $\tau$ between the two rankings for each of the 1000 instances and then report the mean and standard deviation of those 1000 scores in the table. In Table 4, for each of the five combination methods, we compare the similarity between rankings when using BERT versus PetBERT. In Table 5, we compare each of the five combination methods against each other when fixing TM=BERT, whereas in Table 6 we do the same but fix TM=PetBERT.

**Table 4:** Mean (SD) Kendall’s $\tau$ comparing the $\phi$ rankings of BERT vs PetBERT for each of the five combination methods.

Method	Comparison	Mean (SD)
All-Text	BERT vs PetBERT	0.37 (0.23)
WE 25	BERT vs PetBERT	0.81 (0.11)
WE 50	BERT vs PetBERT	0.81 (0.14)
WE 75	BERT vs PetBERT	0.80 (0.18)
Stack	BERT vs PetBERT	0.70 (0.15)
Total	BERT vs PetBERT	0.70 (0.24)

For each entry, we calculate Kendall’s tau for each of the n = 1000 instances and report the mean (SD). WE refers to *Weighted-Ensemble* with $w$ indicating text model weighting. Taking all n = 5000 instances together, we report the mean (SD) Kendall’s Tau under total.

**Table 5:** Mean (SD) Kendall’s $\tau$ comparing the $\phi$ rankings of each of the combination methods against each other when BERT is the text model.

BERT, by method	All-Text	WE 25	WE 50	WE 75
WE 25	0.43 (0.19)			
WE 50	0.50 (0.21)	0.73 (0.16)		
WE 75	0.52 (0.24)	0.56 (0.20)	0.70 (0.19)	
Stack	0.45 (0.19)	0.71 (0.14)	0.64 (0.17)	0.53 (0.20)

Self-comparisons are trivially perfectly correlated ($\tau =1$) and are omitted. For each entry, we calculate Kendall’s tau for each of the n = 1000 instances and report the mean (SD). WE refers to *Weighted-Ensemble* with $w$ indicating text model weighting.

**Table 6:** Mean (SD) Kendall’s $\tau$ comparing the $\phi$ rankings of each of the combination methods against each other when PetBERT is the text model.

PetBERT, by method	All-Text	WE 25	WE 50	WE 75
WE 25	0.31 (0.19)			
WE 50	0.36 (0.21)	0.72 (0.15)		
WE 75	0.41 (0.25)	0.55 (0.19)	0.71 (0.19)	
Stack	0.33 (0.21)	0.66 (0.15)	0.66 (0.17)	0.55 (0.20)

Self-comparisons are trivially perfectly correlated ($\tau =1$) and are omitted. For each entry, we calculate Kendall’s tau for each of the n = 1000 instances and report the mean (SD). WE refers to *Weighted-Ensemble* with $w$ indicating text model weighting.

Following evidence in Fig. 2 of both *All-Text* models focusing almost entirely on two features, we see these models as the most dissimilar to other combination methods with scores between 0.43 and 0.52 for BERT (Table 5) and 0.31 and 0.41 for PetBERT (Table 6). Despite this, they are also dissimilar to each other with a mean $\tau$ of just 0.37 (Table 4), which suggests that even if the remaining features are similarly small in magnitude (from Fig. 2), they are not often in a similar order. For both text models, the two most similar combination method pairs are [*Weighted-Ensemble w = 0.50*, *Weighted-Ensemble w = 0.25*] and [*Weighted-Ensemble w = 0.50*, *Weighted-Ensemble w = 0.75*]. With a shared methodology, similarity is expected: with only the weighting on the prediction changing, it will only be the ordering of the tabular features relative to the text features that differ. Table 4 shows us how much of a difference changing text model has when fixing the combination method, and we see high mean scores of 0.80–0.81 for each of the *Weighted-Ensembles*, indicating a similar ordering of features for a given instance.

### Comparing the two most influential features

**Figure 3:**
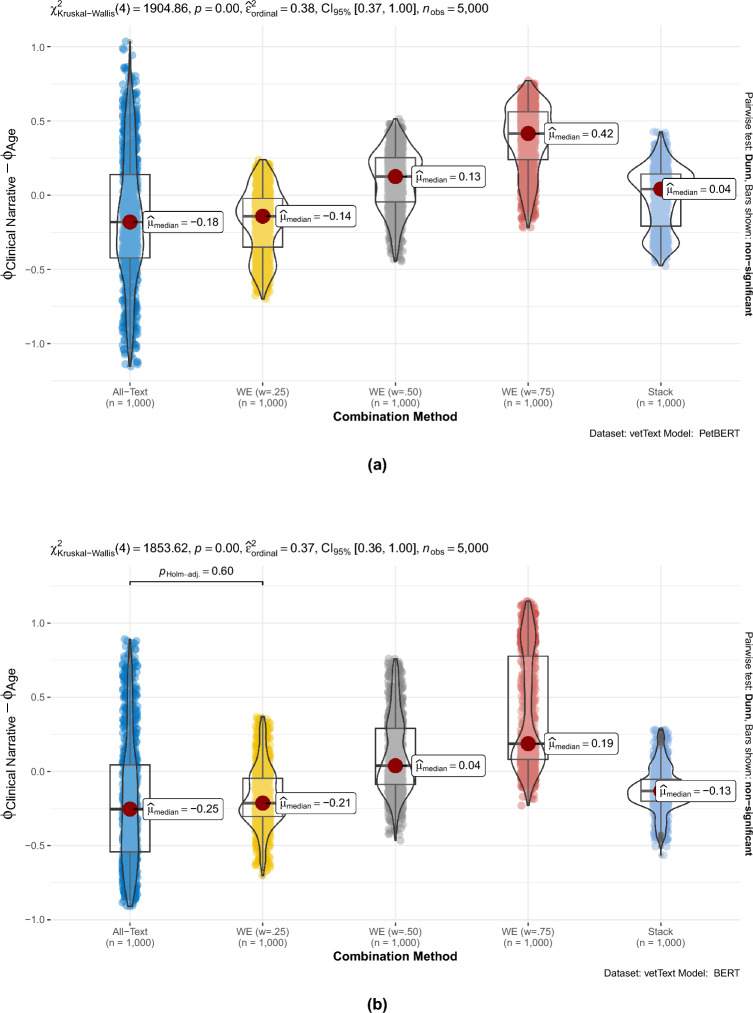
The difference in feature importance (SHAP) between the *clinical narrative* free-text field and *age*, plotted for PetBERT (**a**) and BERT (**b**), for each of the 1000 instances, for each of the five combination methods. The highly significant p-values (both p = 0.00) for both Kruskal–Wallis tests indicate that the median differences for each of the combination methods are not the same; 38% and 37% of the variance in the differences are down to the changing of combination method for (**a**) and (**b**) respectively. Comparing (**a**) and (**b**), we see higher median differences for PetBERT, indicating a higher reliance on the clinical narrative than BERT. Assessing combination methods, we see the preference for the text feature increase as *w*, the text model weighting, increase.

Here, we look at another way of comparing the different models; we aim to get a more general idea of how the two most influential features (*clinical narrative*, a text feature, and *age at consult*, a tabular feature) are treated using each of the five combination methods. For each of the 1000 instances, we examine the difference in feature importance between these two features. In Fig. 3a, we plot the difference between these two for each of the five combination methods when TM=PetBERT and repeat for TM=BERT in Fig. 3b. Once more, we can see greater importance being placed on *clinical narrative* than *age at consult* for PetBERT when compared to BERT, with all combination methods scoring a higher median difference. For *All-Text* experiments, we see far longer tails in the difference distributions than the other methods. This again provides evidence of the importance of both features, differences further away from 0 indicating many cases where *age at consult* is key, *clinical narrative* is not, and vice versa. Furthermore, we also confirm the increased reliance on text features, in this case, *clinical narrative*, with the difference growing more positive as *w* increases from 0.25 through to 0.75.

### Top phrases and tabular values

So far, we have considered all features as a whole, summing SHAP values for individual words to provide an overall score of importance for the entire feature, comparing text features to tabular features. We have seen *clinical narrative,* a text feature, as the most influential in Fig. 2. In a text-only context, one can use the original SHAP library to identify individual word pieces that are the most influential across an entire set of predictions. Using the multimodal SHAP for the first time, we can directly compare individual words to individual tabular feature values. To avoid analysing fragments of words, we set a grouping threshold such that word pieces are grouped into words and phrases. Using the optimal model, PetBERT *Weighted-Ensemble*, w = 0.50, we look at the 1000 instances and find the phrases and tabular values that were the most influential. As a comparison, we also repeat the analysis for BERT *Weighted-Ensemble*, w = 0.50. For those that appear more than once, we take a mean average. The top and bottom five items are found in Table 7, where top and bottom refer to those that contribute the most towards predictions of *alive* and *dead*, respectively. Looking more broadly at the top and bottom 100 phrases and tabular values, we see the tabular feature *age at consult* dominates. For the PetBERT model, of the top 100 entries, all 100 were based on low *age at consult* values and 89 entries from the bottom 100 were based on high *age at consult* values. Similarly in the BERT model, 99 out of the top 100 and 89 of the bottom 100 were from low and high *age at consult* values, respectively. In Fig. 2, we identified that *age at consult* was an important feature, and here we indeed confirmed that older animals are more likely to be found within predictions of *dead* and vice verse for predictions of *alive*. The median age at consultation was 7.1 for the highest and 13.58 for the lowest. The median average for *age at consult* in the dataset was calculated to be 6.29.

**Table 7:** Phrases or instances of tabular features with the highest and lowest SHAP values across all instances for the best performing model, PetBERT *Weighted-Ensemble* w = 0.50, and the BERT equivalent.

SHAP value	Phrase or tabular value	SHAP value	Phrase or tabular value
0.25	Age_at_consult = 0.61	0.25	Age_at_consult = 0.61
0.24	Age_at_consult = 1.55	0.25	Alert and responsive hydration normal.
0.24	Age_at_consult = 0.37	0.24	Age_at_consult = 1.55
0.24	Age_at_consult = 0.93	0.24	Age_at_consult = 0.37
0.24	Age_at_consult = 1.19	0.24	Age_at_consult = 0.93
...	...	...	...
$-0.15$	For older cat	$-0.16$	Hearing with old age
$-0.19$	Of nasal tumour	$-0.18$	Down with age
$-0.23$	Hearing with old age	$-0.20$	QOL etc
$-0.24$	His age	-0.20	Age related?
$-0.28$	Generally slowing down with age	$-0.21$	Some muscle mass loss but

A positive number indicates that the phrase contributes towards a prediction of *alive*, whereas a negative number contributes towards a prediction of *dead*. Left: PetBERT *Weighted-Ensemble* w = 0.50. Right: BERT *Weighted-Ensemble* w = 0.50.

Continuing with our analysis of the top performing PetBERT model (*Weighted-Ensemble* w = 0.50) and its BERT equivalent, we assess which types of phrases are most influential and how they compare to tabular features other than *age at consult*. We remove the dominant tabular feature and examine the new top 100 and bottom 100 lists of phrases and values. To summarise this information, we group each term into 10–12 high-order categories for each list. We count the items in each category and report the seven most populous groups for the top and bottom list, both for PertBERT and BERT. These results are displayed in Table 8, along with examples that typify each group. This representation conveys that the clinical language focused on by each model differs significantly, even if they fall under the same designated category.

**Table 8:** Word and phrases with highest and lowest feature importance (SHAP) values, grouped by high-level category.

Contributing most to a prediction of alive					
BERT Weighted-Ensemble, w =0.50			PetBERT Weighted-Ensemble, w=0.50		
Category	N	Example	Category	N	Example
Symptoms and health conditions	22	“hydration good”, “moulting”, “DUDE normal” “DUDE all ok”	Symptoms and health conditions	18	“spay wound”, “checks over fine”, “No concerns”, “BAR”
Veterinary treatments and procedures	15	“express anal glands”, “skin improving”, “deep oral exam”, “trimmed”	Medications	15	“worming”, “endectrid”, “easecto”, “quantex”
Physical examination findings	11	“ears are fine”, “coat good”, “nothing abnormal detected”, “perineum normal”	Physical examination findings	14	“abdo palp NAD”, “otherwise NAD”, “CE unremarkable”
Medications	8	“advise drops”, “analgesia” “wormer”, “spot on”	Advice given to pet owners	13	”advised joint supplements”, “adv re neuter”, “discussed kc vaccine”
Advice given to pet owners	7	“explained to owner”, “Advised regular bathing”, “bring back if concerned”	Vaccinations	12	“nobivac tricat”, “rhd”, “lepto4”, “DHP/ L4 + KC given”
Vaccination	6	“administered vaccine”, “vaccine”, “booster given”	Weight and body condition score	6	“BCS 5/9 good growth.”, “nice weight”, “28kg”, “8kg”
Pet Behaviour	6	“very well behaved”, “biting”, “not biting, “demean”	Dental conditions and treatments	5	“teeth are great”, “Dentition” “teeth good”, “teeth clean”

Contributing most to a prediction of dead					
Category	N	Example	Category	N	Example
Symptoms and health conditions	25	“muscle mass loss”, “dysuria”, “constipated”, “vomiting”	Diagnoses	24	“mammary tumours”, “Bladder cystitis”, “osteoarthritis”
Age related issues	14	“age-related hearing loss”, “old dog”, “given age”, “getting very old”	Symptoms and health conditions	14	“mobility issues”, “Blind”, “Weak”, “ulcerated”
Food Diet	10	“not eating for 3 days”, “drinking ok”, “not eating well” “been eating more”	Quality of life and euthanasia considerations	13	“euthanase”, “palliative”, “medical management”, “quality of life”
Owner’s observations and concerns	10	“O reports is drinking”, “o aware decline inevitable”, “o concerned coughing”	Medical procedures further testing	10	“bloods”, “biopsy results”, “Ultrasound”, “drain”
Medications	8	“Continue with steroid”, “butorphanol”, “prescription”, “prednisolone”	Age Related Issues	8	“ slowing down with age”, “age related?”, “old age”, “surgery too risky with age”
Weight and body condition score	8	“seems to have lost weight”, “lost weight”, “lost 100 g”	Medications	6	“prednisolone”, “mirtazapine”, “chemo”, “Vivitonin”
Vitals and physical examination findings	7	“Bladder not palpable”, “exam - senile”, “Strong pulses”, “R thyroid slightly enlarged”	Owner’s observations and concerns	6	“o aware decline inevitable”, “doesn’t want investigation”, “o mentioned happier”

[High/low] represents those that contribute most to a prediction of [alive/dead]. Phrases from BERT *Weighted-Ensemble*, *w* = 0.50 and PetBERT *Weighted-Ensemble*, *w* = 0.50 are found on the left and right respectively. PetBERT’s increase in performance can be attributed to its greater likelihood of identifying shorthand or medical terms, as demonstrated by the increased frequency and greater complexity of words in the “Medications” and “Vaccinations” categories. N.B. ”*DUDE*” ’defecating, urinating, drinking and eating’, ”*BAR*” ’bright and responsive’, ”*NAD*” nothing abonroaml detected, ”*CE*” ’clinical examination’, ”*f/w*” flea and wormer treatment, ”*kc*” kennel cough ”nobivac tricat” = vaccine for feline calicivirus, feline herpes virus type 1 and feline panleucopenia virus, ”*rhd*” rabbit haemorrhagic disease vaccine, ”*lepto4*” Canine leptospirosis vaccine, ”*DHP*” distemper, hepatitis (canine adenovirus) and canine parvovirus vaccine, ”*BCS*” ’body condition score’.

**Figure 4:**
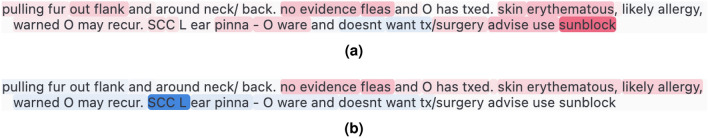
Contrasting explanations for an example where BERT (**a**) was incorrect and PetBERT (**b**) was correct, both *Weitghed-Ensemble*, *w* = 0.50. Words and phrases coloured [red/blue] indicate those that the model found to contribute towards a prediction of [alive/dead]. Both ensembles share an identical tabular model; therefore, we show the subset of the input from the clinical narrative to better exhibit the difference in explanations.

We further illustrate this point with a specific example shown in Fig. 4, once more using *Weighted-Ensemble*, *w* = 0.50. In this case, the true outcome was *alive*; the PetBERT *Weighted-Ensemble* predicted this correctly, whereas the BERT equivalent did not. We see that both text models recognise “no evidence fleas” as a positive sign. Similarly, erythematous—a typically non-serious reddening of the skin—was contributed towards an *alive* prediction for both models. However, the critical difference was that PetBERT identified “SCC L” as shorthand for Squamous Cell Carcinoma, Left (ear) and a clear indicator that this particular animal is not likely to survive. The BERT model did not recognise this as the case and, in fact, regarded “SCC L” as a positive sign for this animal.

## Discussion

An abundance of data lies within the vast volumes of electronic health records collected by initiatives such as SAVSNET. These records extend far beyond textual narratives alone, offering a diversity of modalities to be explored. Nevertheless, the path to harnessing the full potential of these rich datasets is challenging. While immensely powerful, the nature of deep learning frameworks becomes a source of complexity in the context of multimodality predictions. The principal challenge in this endeavour is the innate need for explainability within these frameworks, limiting our ability to extract comprehensive insights from complex AI models and their predictions. This is of paramount importance in the clinical domain, where transparency and interpretability are critical for gaining trust and acceptance among healthcare professionals and regulatory bodies. This paper continues and applies our prior research to this field, allowing us to get insight into a host of multimodal methods for the first time. We use our multimodal masking framework designed to engage in feature masking based on their respective modalities, ensuring uniform and consistent treatment of features, therefore fostering predictability in unimodal and multimodal contexts. This addresses the challenge of generating SHAP explanations for multimodal inputs, extending beyond the traditional unimodal context. In this study, we applied our framework to a text-tabular dataset of EHRs sourced from first-opinion veterinary practices across the United Kingdom to understand the features associated with mortality.

We examined the level of importance assigned to each feature and found a diverse set of preferences between combination methods, but overall, a strong preference for the free-text *clinical narrative* and the tabular feature *age at consult*. For all combination methods tested, PetBERT found an increased relative importance for *clinical narrative* when compared to BERT, a difference most pronounced in the *Stack-Ensembles*. The *Weighted-Ensembles* appeared similar to each other, with no other comparison between combination methods scoring higher than that between the *Weighted Emsembles* *w* = 0.50 and *w* = 0.25. This is consistent with what we expect as the same model structure is used, differing by a linear transformation. More generally, we found that changing the combination method had a greater impact on which features were attended to than changing the text model. For this particular dataset, both the underlying text and tabular models scored similarly well. Therefore, the differences in F1 scores for the ensemble models were also similar despite differing features contributing. A much-reduced importance for other tabular features in the *All-Text* models suggests that information contained in these features, such as a cancer diagnosis in *neoplasms*, is already broadly covered in the free-text *clinical narrative* and is therefore ignored by the text models. However, in the same vein, we suggest that not all information was captured as results in Tables 2 and 3 show that for both text models, *All-Text* was outperformed by *Weighted-Ensemble* *w* = 0.50. The ICD labels represent a broad clinical history of a given animal; therefore, there will be instances where there is an overlap of events within the ICD set and the free-text narrative and other times where the label represents clinical events from many years prior.

Unsurprisingly, there was a notable enhancement in model performance arising from the additional pre-training of PetBERT on 500 million tokens from veterinary clinical narratives when compared to the standard BERT-base model. We observed F1 and accuracy performance improvements of 2% compared to the BERT-base model employing the same evaluation strategy within our best-performing method. While this outcome aligns with our initial expectations, our methodological analysis offers insight into the divergent utilisation of distinct data modalities within the models. To understand the performance of both models on a more granular level, we explored the types of words, phrases and tabular values that were most influential for each model. This was overwhelmingly predominated by *age at consult*. A general and expected trend emerged, suggesting older ages were more likely to die than lower ages. To better discern the difference between BERT and PetBERT, we looked at the words, phrases and tabular values without the presence of *age at consult*. Notably, there were overlaps observed here; for instance, discussions around vaccination were a common theme associated with animals predicted to be alive within the next 28 days. This emphasis between the two models aligns with the inherent logic that one typically would not vaccinate a severely ill animal. Other examples include references to “no concerns” categorised into the “physical examination findings”, which appeared as the third most common category of phrases in both PetBERT and BERT. Phrases such as “other NAD [Nothing Abnormal Detected]” and “CE [Clinical Examination] unremarkable” are unlikely to be used for animals expected to die imminently. Conversely, for words and phrases attributed to an animal approaching death, we observed a shared emphasis on discussions related to symptoms and health conditions. However, the significance of this indicator was more pronounced in the BERT model than in PetBERT. This approach also revealed that PetBERT exhibits a heightened “understanding” across veterinary clinical free-text. This advantage enables PetBERT to interpret the veterinary clinical language associated with these subject matters more effectively than regular English, on which BERT was initially trained. Distinctly, PetBERT selected more definitive diagnoses as a more significant indicator, such as in “mammary tumours”. Overall, words and phrases around cancers and mass growths emerged as noteworthy indicators in both models, although more so in PetBERT. Although both models identified signs of vaccination as a positive indication, the words and phrases differed. PetBERT selected specific vaccination names such as “lepto4” and “nobivac tricat”, whereas BERT used more generalised terms such as “booster” and “vaccine”. When thinking about the generalised corpora that BERT was trained on, there is a frequent theme where veterinary-specific terminology is not well understood, but phrases shared with human clinical medicine are present. Another example is within the “medications” category, BERT’s utilisation of drug names “steroids”, “butorphanol”, and “prednisolone” are all authorised drugs used frequently in human medicine. However, drugs such as “Vivitonin”, which was utilised by PetBERT, are authorised solely for dogs in the UK. Increased comprehension of phrases pertinent to diagnostic diseases, drug names, and diagnostic tests could attest to PetBERT’s superior clinical proficiency.

The framework we have employed is fundamentally underpinned by SHAP and transformers, both of which are computationally expensive. This computational burden can lead to prolonged processing times, potentially limiting the scalability of our approach, especially when working with larger datasets or in real-time clinical settings. In the context of *All-Text*, a single style of string template was the exclusive choice. In future investigations, exploring the impact of diverse template styles on explanations could be beneficial. The initial study developed a classifier that identified animals that have died with an F1 score exceeding 98.3%. Both the previous study and this study characterised the outputs for use within the prediction of mortality risk modelling. Therefore, it is likely that some data used within this study was incorrectly misclassified. While this level of misclassification is unlikely to impact the overall findings substantially, it is a point of consideration when interpreting individual predictions or decisions based on the model’s output. Furthermore, the dataset used in this study was sourced from participating veterinary practices. Consequently, the findings presented here may only partially represent the broader UK companion animal population. As the national coverage of participating practices within the Small Animal Veterinary Surveillance Network (SAVSNET) expands, these issues of coverage bias may be mitigated. Throughout our analysis, we have used the F1 score as the principal measure for a model’s quality due to it being a balance between precision and recall. We believe this provides models which are well-rounded, however, we note that some may prefer to optimise for precision, recall or another metric entirely which may affect which experiments are the most effective.

To conclude, this study investigated the complex dynamics governing the interaction between deep learning models and data modalities in the context of veterinary clinical EHRs. The findings suggest that the changing modality combination method has a more substantial influence on which features models find important, whereas both text models in this study tended to rank similar features as important. Additionally, PetBERT, having undergone additional pre-training, demonstrated enhanced comprehension of phrases related to cancer, drug names, and diagnostic tests, suggesting its superior proficiency in veterinary clinical language compared to BERT. The study highlights the capacity of language models to extract valuable insights from clinical narratives, providing contextual factors that inform predictions regarding animal well-being. The comparative analysis of both modalities within a uniform framework has significantly enabled the comprehension and interpretation of the overall model prediction and enabled a per-input feature comparison, regardless of whether that be a text or tabular value.

## Data Availability

The datasets generated and analysed during the current study are not publicly available due to privacy concerns around sensitive owners’ information but are available from the corresponding author on reasonable request.
